# MLKL-mediated necroptosis is a target for cardiac protection in mouse models of type-1 diabetes

**DOI:** 10.1186/s12933-022-01602-9

**Published:** 2022-08-27

**Authors:** Ting Cao, Rui Ni, Weimin Ding, Xiaoyun Ji, Lan Li, Guangneng Liao, Yanrong Lu, Guo-Chang Fan, Zhuxu Zhang, Tianqing Peng

**Affiliations:** 1 Institutes of Biology and Medical Sciences, Soochow University, Suzhou, China; 2 Lawson Health Research Institute, London Health Sciences Centre, VRL 6th Floor, A6-140, 800 Commissioners Road, London, ON N6A 5W9 Canada; 3 Department of Pathology and Laboratory Medicine, Western University, London, ON Canada; 4 Key Laboratory of Transplant Engineering and Immunology, NHFPC, and Regenerative Medicine Research Center, West China Hospital, Sichuan University, Chengdu, China; 5 Department of Pharmacology and Systems Physiology, University of Cincinnati College of Medicine, Cincinnati, OH USA; 6 Department of Medicine, Western University, London, ON Canada

**Keywords:** MLKL, RIPK3, Necroptosis, Cardiomyocytes, Diabetes, Heart

## Abstract

**Background:**

Cardiomyocyte death contributes to cardiac pathology of diabetes. Studies have shown that the RIPK3/MLKL necroptosis signaling is activated in diabetic hearts. Deletion of RIPK3 was reported to attenuate myocardial injury and heart dysfunction in streptozocin (STZ)-induced diabetic mice, suggesting a potential role of necroptosis in diabetic cardiomyopathy. This study characterized cardiomyocyte necroptosis in diabetic hearts and investigated whether MLKL-mediated necroptosis is a target for cardiac protection in diabetes.

**Methods:**

Type 1 diabetes was induced in RIPK3 knockout, MLKL knockout and wild-type mice. Akita Type-1 diabetic mice were injected with shRNA for MLKL. Myocardial function was assessed by echocardiography. Immuno-histological analyses determined cardiomyocyte death and fibrosis in the heart. Cultured adult mouse cardiomyocytes were incubated with high glucose in the presence of various drugs. Cell death and phosphorylation of RIPK3 and MLKL were analysed.

**Results:**

We showed that the levels of phosphorylated RIPK3 and MLKL were higher in high glucose-stimulated cardiomyocytes and hearts of STZ-induced type-1 diabetic mice, akita mice and type-1 diabetic monkeys when compared to non-diabetic controls. Inhibition of RIPK3 by its pharmacological inhibitor or gene deletion, or MLKL deletion prevented high glucose-induced MLKL phosphorylation and attenuated necroptosis in cardiomyocytes. In STZ-induced type-1 diabetic mice, cardiomyocyte necroptosis was present along with elevated cardiac troponin I in serum and MLKL oligomerization, and co-localized with phosphorylated MLKL. Deletion of RIPK3 or MLKL prevented MLKL phosphorylation and cardiac necroptosis, attenuated serum cardiac troponin I levels, reduced myocardial collagen deposition and improved myocardial function in STZ-injected mice. Additionally, shRNA-mediated down-regulation of MLKL reduced cardiomyocyte necroptosis in akita mice. Interestingly, incubation with anti-diabetic drugs (empagliflozin and metformin) prevented phosphorylation of RIPK3 and MLKL, and reduced cell death in high glucose-induced cardiomyocytes.

**Conclusions:**

We have provided evidence that cardiomyocyte necroptosis is present in diabetic hearts and that MLKL-mediated cardiomyocyte necroptosis contributes to diabetic cardiomyopathy. These findings highlight MLKL-mediated necroptosis as a target for cardiac protection in diabetes.

**Supplementary Information:**

The online version contains supplementary material available at 10.1186/s12933-022-01602-9.

## Background

Cardiovascular complications of diabetes have been recognized as the leading cause of morbidity and mortality in the diabetic population. Heart failure has emerged as the most common initial cardiac complication of diabetes [1]. The diabetic population poses a marked preponderance to developing heart failure following myocardial infarction [2–4]. In addition, myocardial dysfunction has been described in diabetes in the absence of coronary artery disease and other cardiovascular diseases, a condition described as diabetic cardiomyopathy, which involves structural, functional and metabolic changes in the heart [5]. However, the pathogenesis of diabetic cardiomyopathy has not been fully understood. Diabetic conditions have been reported to induce different forms of cardiomyocyte death [6]. Loss of cardiomyocytes is the fundamental pathological process triggering the cascade of adverse myocardial remodeling that leads to heart failure [7]. Thus, inhibition of cardiomyocyte death may represent an effective strategy to reduce cardiac complications of diabetes.

Cardiomyocyte apoptosis and pyroptosis have been implicated in diabetic cardiomyopathy [8–15]. However, experimental evidence is limited regarding the role of other forms of cell death in diabetic cardiomyopathy. Necroptosis has emerged as an important mechanism in myocardial infarction [16] and doxorubicin-induced cardiotoxicity [17]. Necroptosis is regulated by receptor interacting protein kinase 1 (RIPK1), RIPK3 and mixed lineage kinase domain-like pseudokinase (MLKL). RIPK1 and RIPK3 interact and cross-phosphorylate. RIPK3 then phosphorylates MLKL, which allows its recruitment to the inner cell membrane to form a lethal pore, inducing membrane rupture [18–21]. It has been recently reported that RIPK3 and MLKL necroptosis signaling is activated in cardiomyocytes and hearts under diabetic conditions [22–26]. A recent study showed that deletion of RIPK3 attenuated serum makers of myocardial injury and heart dysfunction in streptozocin (STZ)-induced diabetic mice [27]. These previous studies have suggested a potential role of necroptosis in diabetic cardiomyopathy. However, cardiomyocyte necroptosis has not been well characterized in diabetic hearts. Moreover, although MLKL is the most well-established substrate of RIPK3 in executing necroptosis in many cell types, RIPK3 has been also reported to mediate cardiomyocyte necroptosis through MLKL-independent mechanisms in myocardial infarction, doxorubicin-induced cardiotoxicity and tunicamycin-induced endoplasmic reticulum stress [17, 28]. Nevertheless, the role of MLKL in cardiomyocyte necroptosis and heart dysfunction during diabetes remains elusive.

In this study, we characterized cardiomyocyte necroptosis in diabetic hearts and examined the role of MLKL-mediated necroptosis in diabetic cardiomyopathy. Additionally, the effect of anti-diabetic drugs (empagliflozin and metformin) was determined in high glucose-induced necroptosis.

## Methods

### Animals

This investigation conforms to the Guide for the Care and Use of Laboratory Animals published by the US National Institutes of Health (NIH Publication, 8th Edition, 2011). All mouse experimental procedures were approved by the Animal Use Subcommittee at the University of Western Ontario, Canada (protocol number: 2021-054), and Soochow University, China (protocol number: 2017-0043). Breeding pairs of C57BL/6 mice and akita type-1 diabetic mice were purchased from the Jackson Laboratory. RIPK3 knockout mice were kindly provided by Dr. Xiaodong Wang (National Institute of Biological Sciences, Beijing, China) and MLKL knockout mice were purchased from the Cyagen Biosciences (Suzhou, China). All animals were housed in a temperature- and humidity-controlled facility on a 12-h light and dark cycle with water and food ad libitum.

All rhesus macaque experiments were approved by the Institutional Animal Care and Use Committee of Experimental Animal Center, West China Hospital, Sichuan University, China (protocol number: 2014004A).

### Experimental protocols

Type-1 diabetes was induced in adult mice (male, 2 months old) by intraperitoneal injections of streptozocin (STZ, 50 mg/kg/day, Sigma, Oakville, ON, Canada) for 5 consecutive days [29]. Seventy-two hours after the last injection of STZ, whole blood was obtained from the mouse tail vein, and random glucose levels were measured using the OneTouch Ultra 2 blood glucose monitoring system (LifeScan, Inc., Malvern, PA, USA). The mice were considered diabetic and used for the study only if their blood glucose levels were ≥ 15 mmol/L 72 h after STZ injection, whereas citrate buffer-injected mice were used as normoglycemic controls (blood glucose < 12 mmol/L).

GSK’872 (5 mg/kg/day, i.p.) or vehicle was administrated to mice (10 mice in each group) right after the last injection of STZ for a total of 2 months [30]. The plasmid DNA containing MISSION^®^shRNA for MLKL (*p*shMLKL, 60 µg, Sigma, Oakville, ON, Canada) or an empty plasmid was injected into Akita mice (male, 2 months old, 6 mice in each group) through tail veins with GenJet™ Plus DNA in vivo transfection reagent following the manufacturer’s instructions (SignaGen^®^ Laboratories, Frederick, MD, USA). Three days later (day 3), a second dose of *p*shMLKL or empty plasmid injection was given to the same mice. Mice were then killed after 2 days of last plasmid injection (day 5), and their heart tissues and blood were collected for further analyses.

Six rhesus macaques (male at age 3–5 years) received a single dose of STZ (80 mg/kg, Sigma-Aldrich, Shanghai, China) intravenously to induce diabetes as previously described [31]. Insulin was used to maintain the fasting blood glucose level at 15–20 mmol/L in all diabetic rhesus macaques for 7–8 years. Six sham rhesus macaques were fed a standard diet twice per day. Their heart tissues were collected for western blot analysis.

### Echocardiography

Mice were anesthetized with inhaled isoflurane (1%) and imaged using a 40 MHz linear array transducer attached to a preclinical ultrasound system (Vevo 2100, FUJIFILM Visual Sonics, Toronto, ON, Canada) with a nominal in-plane spatial resolution of 40 μm (axial) × 80 μm (lateral). M-mode and 2-D parasternal short-axis scans (133 frames/s) at the level of the papillary muscles were employed to assess changes in left ventricle (LV) end-systolic inner diameter, LV end-diastolic inner diameter, LV posterior wall thickness in end-diastole and end-systole, ejection fraction (EF) and fractional shortening (FS). To assess diastolic function, pulsed wave Doppler measurements of maximal early (E) and late (A) transmittal velocities in diastole were obtained in the apical view with a cursor at mitral valve inflow.

### Cell membrane permeability to Evans blue dye (EBD)

EBD (Sigma, Oakville, Ontario, Canada) was dissolved in saline and injected into mice (100 mg/kg body weight, i.p.). Twenty-four hours later, the mice were euthanized by cervical dislocation under anesthesia with a mixture of ketamine (100 mg/kg)/xylazine (5 mg/kg, i.p., Sigma, Oakville, Ontario, Canada) after ensuring that they did not respond to a needle prick of the skin. The hearts were harvested, embedded in optimal cutting temperature (OCT) compound (Sakura, VWR International, Mississauga, ON, Canada), snap frozen in liquid nitrogen, and cut into 5-μm cryosections. Nuclei were stained using Hoechst 33342 following the manufacturer’s instructions (Thermo Fisher Scientific Inc., Burlington, Ontario, Canada). EBD uptake (red) was visualized under a fluorescence microscope as described previously [32]. Cell death was quantified as the ratio of nuclei in EBD positive cells over total nuclei from 3 different cross-sections for each heart.

### Immuno-fluorescence staining

Frozen sections of heart tissues were incubated with primary antibodies against phosphorylated MLKL and cardiac troponin I (New England Biolabs Ltd., Whitby, Ontario, Canada), and then secondary antibodies (Donkey anti-rabbit IgG antibody conjugated with fluorescein isothiocyanate [FITC], Santa Cruz Biotechnology Inc., Dallas, TX, USA, or donkey anti-rabbit IgG secondary antibody conjugated with Alexa Fluor Plus 405,Thermo Fisher Scientific Inc., Burlington, Ontario, Canada) as described previously [33]. Normal rabbit IgG (New England Biolabs Ltd., Whitby, Ontario, Canada) was used as a negative control for anti-phosphorylated MLKL antibody and anti-cardiac troponin I. Cell membrane was stained by FITC-conjugated wheat germ agglutinin (WGA, Sigma Aldrich, Oakville, Ontario, Canada) as described previously [29]. Signals were obtained using a fluorescence microscope or confocal microscope.

### Determination of collagen deposition

Heart tissues were fixed in 4% paraformaldehyde (Sigma, Oakville, Ontario, Canada) at 4 °C for 48 h and then routinely processed, wax-embedded and sectioned. After processing, the tissue section (5 μm thick) were stained with a saturated solution of picric acid containing 1% Sirius red (Sigma, Oakville, Ontario, Canada) for collagen deposition measurement [29, 34].

### Measurement of CK-MB and cardiac troponin I

CK-MB levels in sera were determined using an assay kit from BBI Life Sciences Corporation (Shanghai, China) and serum cardiac troponin I levels were measured using a commercially available kit from Biomatik Corporation (Kitchener, Ontario, Canada) following the manufacturer’s instructions, respectively.

### Adult mouse cardiomyocyte isolation and cultures

Adult mouse ventricle cardiomyocytes were isolated from RIPK3 knockout, MLKL knockout and wild-type mice (C57BL/6) and cultured as described in our recent studies [11, 35].

### Western blot analysis

Heart tissues or cardiomyocytes were homogenized and subjected to SDS–polyacrylamide gel electrophoresis. After the proteins were transferred to PVDF membranes, western blot analysis determined the protein levels of total and phosphorylated RIPK3, MLKL and adenosine 5’ monophosphate-activated protein kinase (AMPK) using their specific antibodies (1: 1000 dilution, New England Biolabs Ltd., Whitby, Ontario, Canada). Horseradish peroxidase (HRP)-conjugated goat anti-mouse IgG (H + L) secondary antibody was purchased from BOSTER Biological Technology (catalog number: BA1051, Wuhan, China,), and HRP-conjugated goat anti-rabbit IgG (H + L) was purchased from Jackson ImmunoResearch (catalog number: 136080, West Grove, PA, USA,).

MLKL oligomerization was determined as previously described [36].

### Caspase-3 activity

Caspase-3 activity in cardiomyocyte lysates was measured using a Caspase-3 Fluorescence Assay Kit (Biomol Research Laboratories, Inc., Plymouth, PA, USA) following the manufacturer’s instructions.

### Assessment of cell death

Necrosis was determined in cardiomyocytes by Propidium Iodide (PI) staining assay and nuclei were stained with Hoechst 33342 following the manufacturer’s instructions (Thermo Fisher Scientific Inc., Burlington, Ontario, Canada). Cell death (apoptosis and necrosis) was assessed by Annexin V staining assay (Thermo Fisher Scientific Inc., Burlington, Ontario, Canada) following the manufacturer’s instructions.

The levels of lactate dehydrogenase (LDH) in culture medium were measured to determine cell injury or death using a commercially available kit (Takara Bio USA, Inc., San Jose, CA, USA) following the manufacturer’s instructions.

### Statistical analysis

The data are expressed as the mean ± SD. Student’s *t* test was employed to compare data between two groups. ANOVA followed by the Newman-Keuls test was performed for multi-group comparisons. A *P* value of less than 0.05 was considered significant.

## Results

### The levels of phosphorylated RIPK3 and MLKL protein are elevated in high glucose-stimulated cardiomyocytes and type-1 diabetic hearts

**Fig. 1:**
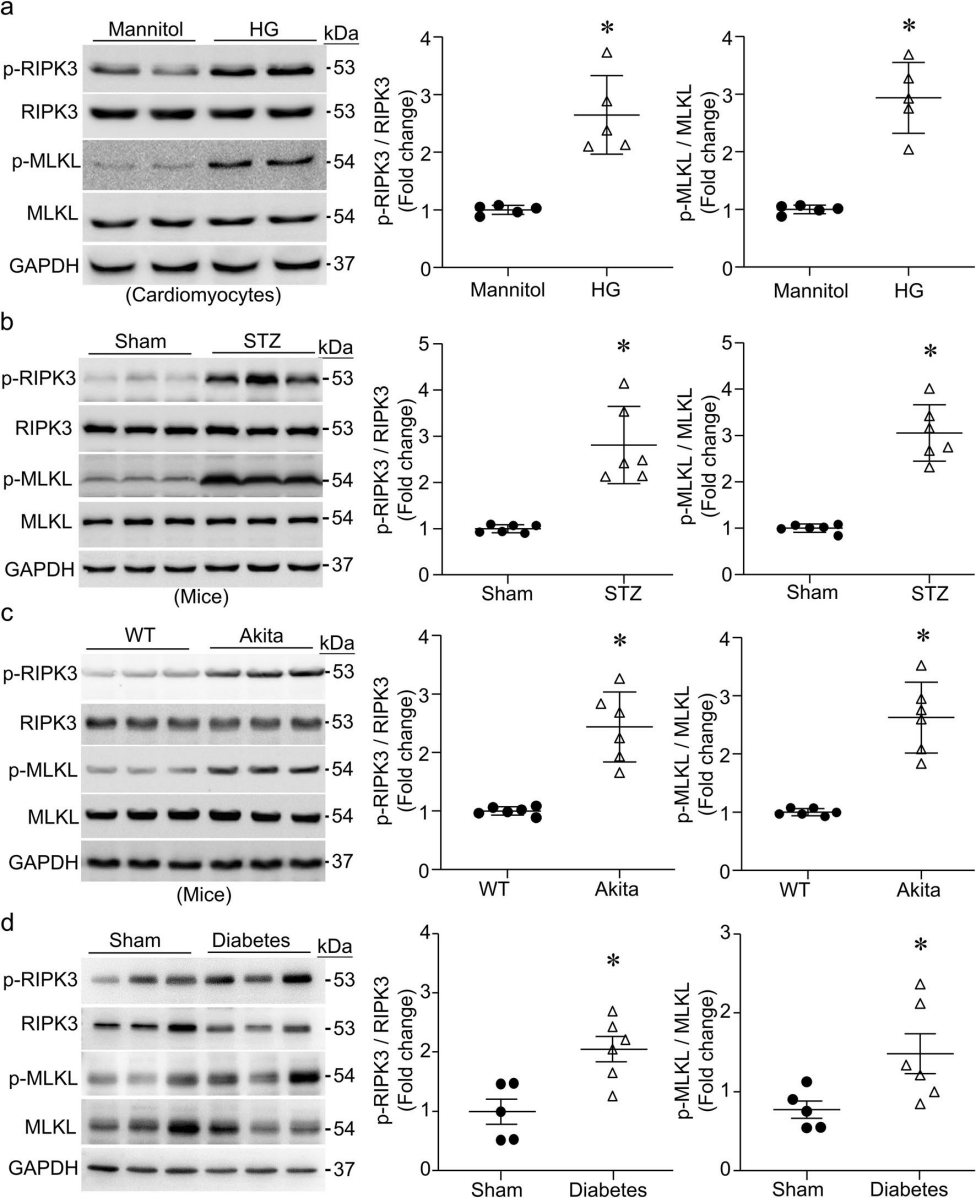
Phosphorylation of RIPK3 and MLKL. **a** Adult mouse cardiomyocytes were incubated with high glucose or mannitol (25 mmol/L) for 24 h. The levels of phosphorylated RIPK3 (p-RIPK3) and MLKL (p-MLKL) were determined by western blot analysis. Left panel: representative western blots for p-RIPK3, p-MLKL and their total proteins as well as GAPDH; Right two panels: quantification of p-RIPK3 and p-MLKL relative to their total proteins, respectively. **b**–**d** The levels of p-RIPK3 and p-MLKL in hearts of streptozotocin (STZ)-injected mice (**b**), Akita mice (**c**) and monkeys (**d**) were analyzed by western blot. Left panel: representative western blots from 3 out of 5–6 different hearts in each group for p-RIPK3 and p-MLKL, total RIPK3, total MLKL and GAPDH; Right two panels: quantification of p-RIPK3 and p-MLKL (WT: wild-type). Data are mean ± SD, n = 5–6. * *P* < 0.05 versus Mannitol, Sham or WT (t test)

Adult mouse cardiomyocytes were incubated with high glucose (25 mmol/L) or mannitol (25 mmol/L) as an osmotic control for 24 h. Western blot analysis revealed higher levels of phosphorylated RIPK3 and MLKL in high glucose- compared with mannitol-stimulated cardiomyocytes (Fig. 1a). In a mouse model of STZ-induced type-1 diabetes, the levels of phosphorylated RIPK3 and MLKL protein were higher in diabetic hearts after 2 months of last STZ injection when compared to sham mice (Fig. 1b). Similarly, akita type-1 diabetic mice had higher levels of phosphorylated RIPK3 and MLKL in the heart relative to those in sham animals (Fig. 1c). However, the total protein levels of RIPK3 and MLKL were similar between sham and diabetes groups.

To explore the translational significance of the above observations, we determined the levels of phosphorylated RIPK3 and MLKL in heart tissues of STZ injection-induced type-1 diabetic rhesus monkeys. As shown in Fig. 1d, type-1 diabetic monkey hearts exhibited higher levels of phosphorylated RIPK3 and MLKL protein compared with sham monkeys. These results indicate that diabetic conditions activate RIPK3 and MLKL necroptosis signaling in the heart.

### Inhibition of RIPK3 prevents MLKL phosphorylation and necroptosis in high glucose-induced cardiomyocytes

**Fig. 2:**
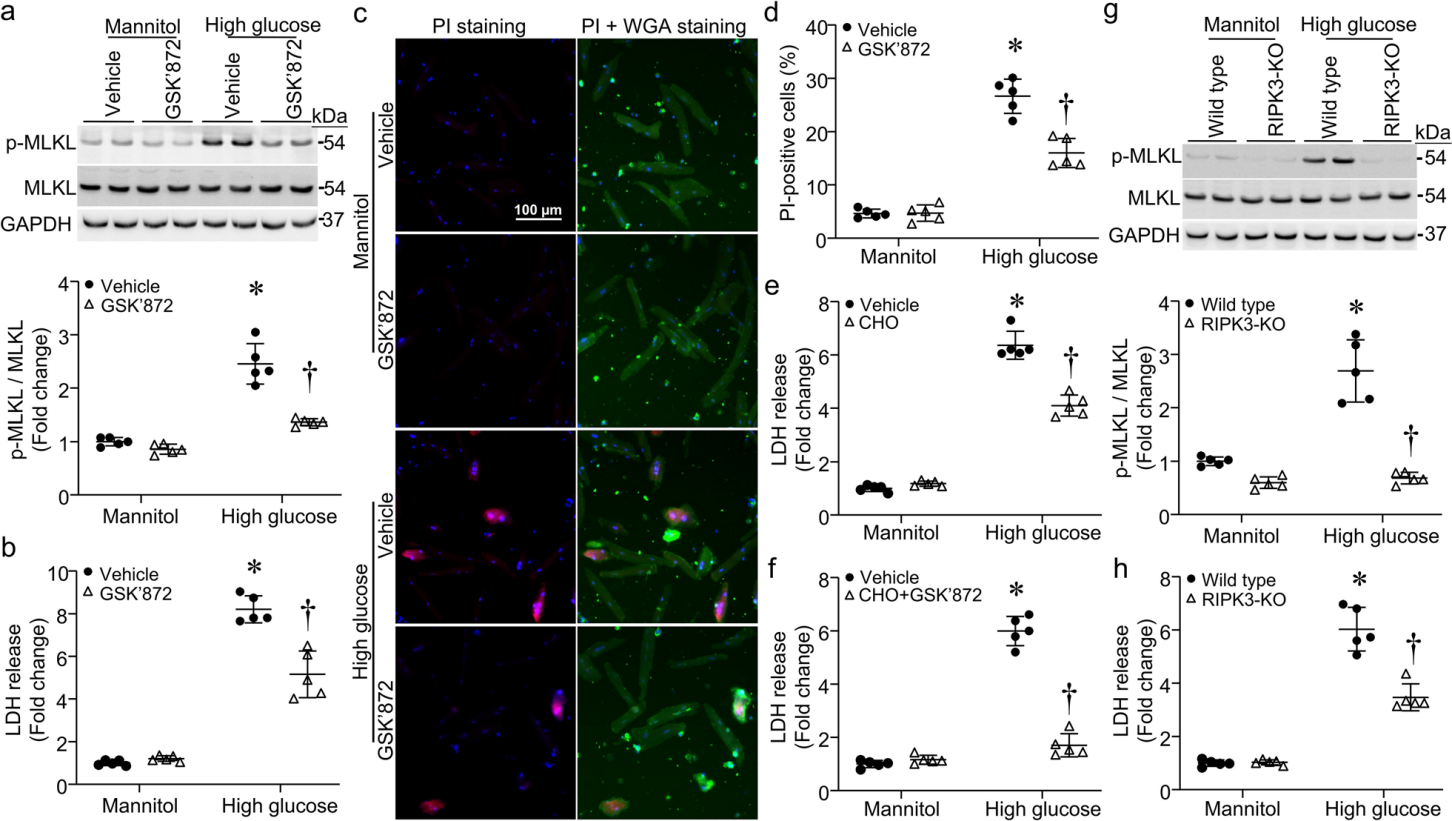
Effects of RIPK3 inhibition on cell death in cardiomyocytes. Adult mouse cardiomyocytes isolated from RIPK3 knockout (RIPK3-KO) and wild-type mice (WT) were incubated with high glucose or mannitol (25 mmol/L) in the presence of GSK’872 and CHO alone or in combination for 24 h. **a** The levels of phosphorylated MLKL were determined by western blot analysis. Upper panel: a representative western blot for phosphorylated MLKL (p-MLKL), total MLKL and GAPDH; Bottom panel: quantification of p-MLKL relative to total MLKL. **b** LDH was measured in culture medium. **c** Necrotic cell death was assessed using PI staining. A representative micro-photograph for PI staining positive cells (red), nucleus Hoechst33342 staining (blue) and FITC-WGA staining for cell membrane (green). **d** Quantification of necrotic cell death. **e** and **f** LDH was measured in culture medium. **g** The phosphorylated levels of MLKL were determined in WT and RIPK3-KO cardiomyocytes. Upper panel: a representative western blot for p-MLKL, total MLKL and GAPDH; Bottom panel: quantification of p-MLKL relative to total MLKL. **h** LDH was measured in culture medium. Data are mean ± SD, n = 5 different cultures. *P < 0.05 versus Mannitol + Vehicle or Mannitol + WT and ^†^ *P* < 0.05 versus HG + Vehicle or HG + WT (ANOVA)

To determine the role of RIPK3 in MLKL phosphorylation and necroptosis, we incubated cardiomyocytes with high glucose or mannitol in combination with RIPK3 inhibitor GSK’872 (5 µmol/L) or vehicle for 24 h. Western blot analysis showed that GSK’872 prevented high glucose-induced MLKL phosphorylation (Fig. 2a). The LDH release assay revealed that high glucose induced cardiomyocyte death (Fig. 2b), which was confirmed by PI staining assay (Fig. 2c). GSK’872 treatment significantly reduced LDH release and PI staining positive cells in high glucose-stimulated cardiomyocytes (Fig. 2b, c, d). These results suggest that RIPK3 mediates high glucose-induced cardiomyocyte necroptosis. High glucose was also reported to induce apoptosis in cardiomyocytes. In line with this, we showed that incubation with caspase-3 inhibitor, Ac-DEVD-CHO (5 µmol/L, VWR International, Mississauga, Ontario, Canada), attenuated high glucose-induced LDH release (Fig. 2e), supporting the involvement of apoptosis. It is known if apoptotic cells are not cleared in a timely manner they progress to secondary necrosis [37]. Since inhibition of RIPK3 or caspase-3 partially prevented cell death in high glucose-stimulated cardiomyocytes, it is possible that necrosis might be secondary to apoptosis. To exclude such possibility, we simultaneously inhibited necroptosis and apoptosis by GSK’872 and Ac-DEVD-CHO, respectively. Co-incubation with GSK’872 and Ac-DEVD-CHO completely inhibited high glucose-induced LDH release in cardiomyocytes (Fig. 2f), indicating that high glucose induces both necroptosis and apoptosis.

To verify the role of RIPK3, we used RIPK3 knockout cardiomyocytes. As shown in Fig. 2g, h, high glucose increased the levels of phosphorylated MLKL in wild-type but not in RIPK3 knockout mouse cardiomyocytes, and high glucose-induced LDH release was much less in RIPK3 knockout compared with wild-type mouse cardiomyocytes. Taken together, these results suggest that RIPK3-mediated necroptosis is associated with MLKL activation in cardiomyocytes under diabetic conditions.

### Cardiomyocyte necroptosis is induced in a mouse model of STZ-induced type-1 diabetes

**Fig. 3:**
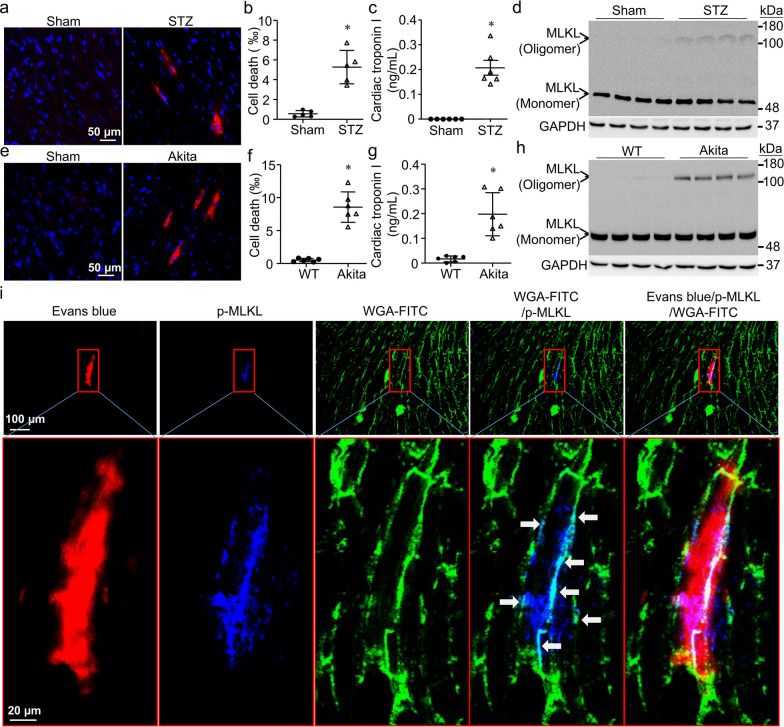
Characterization of cardiomyocyte necroptosis in the heart. **a** and **e** Necrotic cell death was assessed by Evans blue staining assay. Representative micro-photographs show Evans blue stained necrotic cell death (red) in hearts of STZ-injected mice and Akita mice but not in Sham animals. Nuclei were stained blue by Hoechst33342. **b** and **f** Quantification of Evans blue stained necrotic cell death in the heart. **c** and **g** Serum cardiac troponin I. **d** and **h** MLKL oligomerization. Representative western blot from 4 different hearts in each group shows the presence of MLKL oligomerization in STZ-injected and Akita but not Sham mouse hearts. **i** Confocal microscopy. Representative micro-photograph indicates that Evans blue stained necrotic cells (red) are presented with phosphorylated MLKL (p-MLKL, blue), and that punctate staining of p-MLKL co-localizes with WGA-FITC (cyan). Data are mean ± SD, n = 5–6. * *P* < 0.05 versus Sham or WT (t test)

Although activation of RIPK3 and MLKL necroptosis signaling has recently been reported in diabetic hearts [22–27], direct evidence of cardiomyocyte necroptosis is lacking in the heart. To characterize cardiomyocyte necroptosis in the heart in situ, we first determined necrosis in the hearts of STZ-induced type-1 diabetic mice. The Evans blue assay showed necrotic cells in diabetic but not sham mouse hearts (Fig. 3a, b). We then localized necrotic cells in cardiomyocytes of STZ-injected mice by the immuno-fluorescence assay using antibody against cardiac troponin I (Additional file 1: Fig. S1). The presence of cardiomyocyte necrosis in diabetic mice was correlated with an increase in serum cardiac troponin I, a well-established biomarker of myocardial injury (Fig. 3c). In addition to an increase in phosphorylated RIPK3 and MLKL levels (Fig. 1b), we further analyzed MLKL oligomerization in heart tissues by western blot. As shown in Fig. 3d, MLKL oligomer was observed in STZ-injected but not sham mouse hearts, indicating that diabetes induces MLKL oligomerization in the heart. In contrast, MLKL monomer was comparable between sham and STZ-injected mouse hearts. Similarly, akita type-1 diabetic mice had more cardiomyocyte necroptosis, higher serum cardiac troponin I levels and MLKL oligomerization compared to those in their non-diabetic littermate controls (Fig. 3e, f, g, h). These results strongly indicate cardiomyocyte necroptosis in type-1 diabetic mouse hearts.

To provide in situ evidence supporting cardiomyocyte necroptosis, we determined phosphorylated MLKL in diabetic hearts by the immuno-fluorescence assay using antibody against phosphorylated MLKL. As shown in Fig. 3i and Additional file 1: Fig. S2, necroptosis in cardiomyocytes was identified by double staining of necrosis (red color by EBD) and phosphorylated MLKL (blue in Fig. 3i or green color in Additional file 1: Fig. S2). Notably, confocal microscopy revealed co-localization of punctate staining of phosphorylated MLKL and cell membrane staining by FITC-conjugated WGA (cyan color), suggesting MLKL membrane translocation (Fig. 3i). In contrast, no signal was detected when anti-phosphorylated MLKL antibody was replaced with normal rabbit IgG (Additional file 1: Fig. S2). These results provide first in situ evidence that diabetes induces cardiomyocyte necroptosis, which is association with MLKL activation.

### Inhibition of RIPK3 prevents MLKL phosphorylation and necroptosis, and reduces heart dysfunction in mouse models of STZ-induced type-1 diabetes

**Fig. 4:**
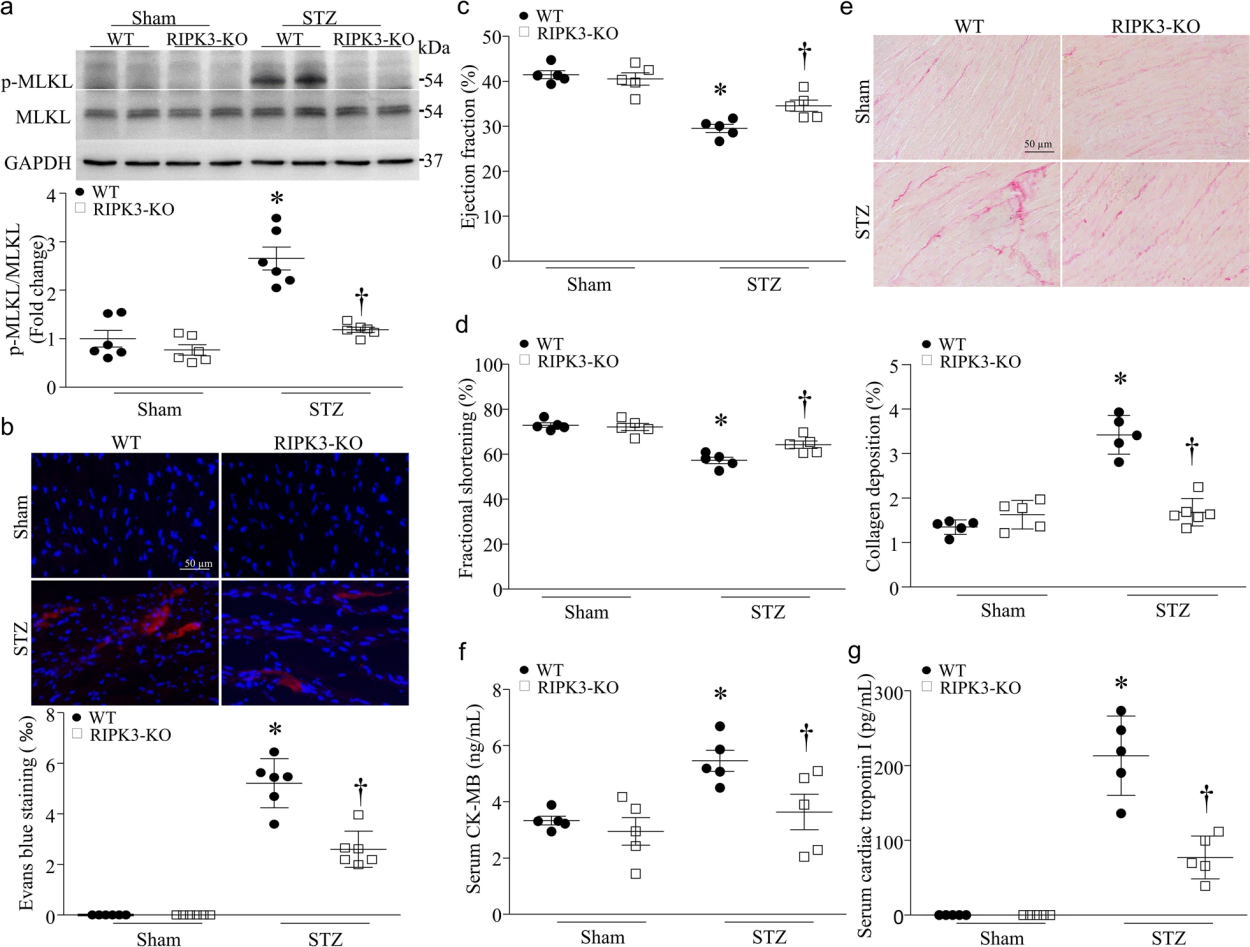
Effects of RIPK3 knockout in streptozocin (STZ)-induced diabetic hearts. RIPK3 knockout (RIPK3-KO) and wild-type mice (WT) were rendered diabetic by STZ injection. **a** The levels of phosphorylated MLKL (p-MLKL) were analyzed by western blot in the heart. Upper panel: Upper panel: a representative western blot from 2 out of 6 different hearts in each group for p-MLKL and total MLKL; Bottom panel: quantification of p-MLKL relative to total MLKL. **b** Necrotic cell death in the heart was determined by Evans blue staining assay. Upper panel: representative micro-photograph for necrotic cells in the heart; Bottom panel: quantification of necrotic cell death. **c** and **d** Echocardiographic analysis for myocardial function. **e** Myocardial fibrosis was assessed by collagen deposition in the heart. Upper panel: representative micro-photograph for collagen deposition in the heart (red); Bottom panel: quantification of collagen deposition. **f** and **g** Biomarkers of myocardial injury: serum CK-MB (**f**) and cardiac troponin I (**g**). Data are mean ± SD, n = 5–6. *P < 0.05 versus Sham + WT and ^†^*P* < 0.05 versus STZ + WT (ANOVA)

**Fig. 5:**
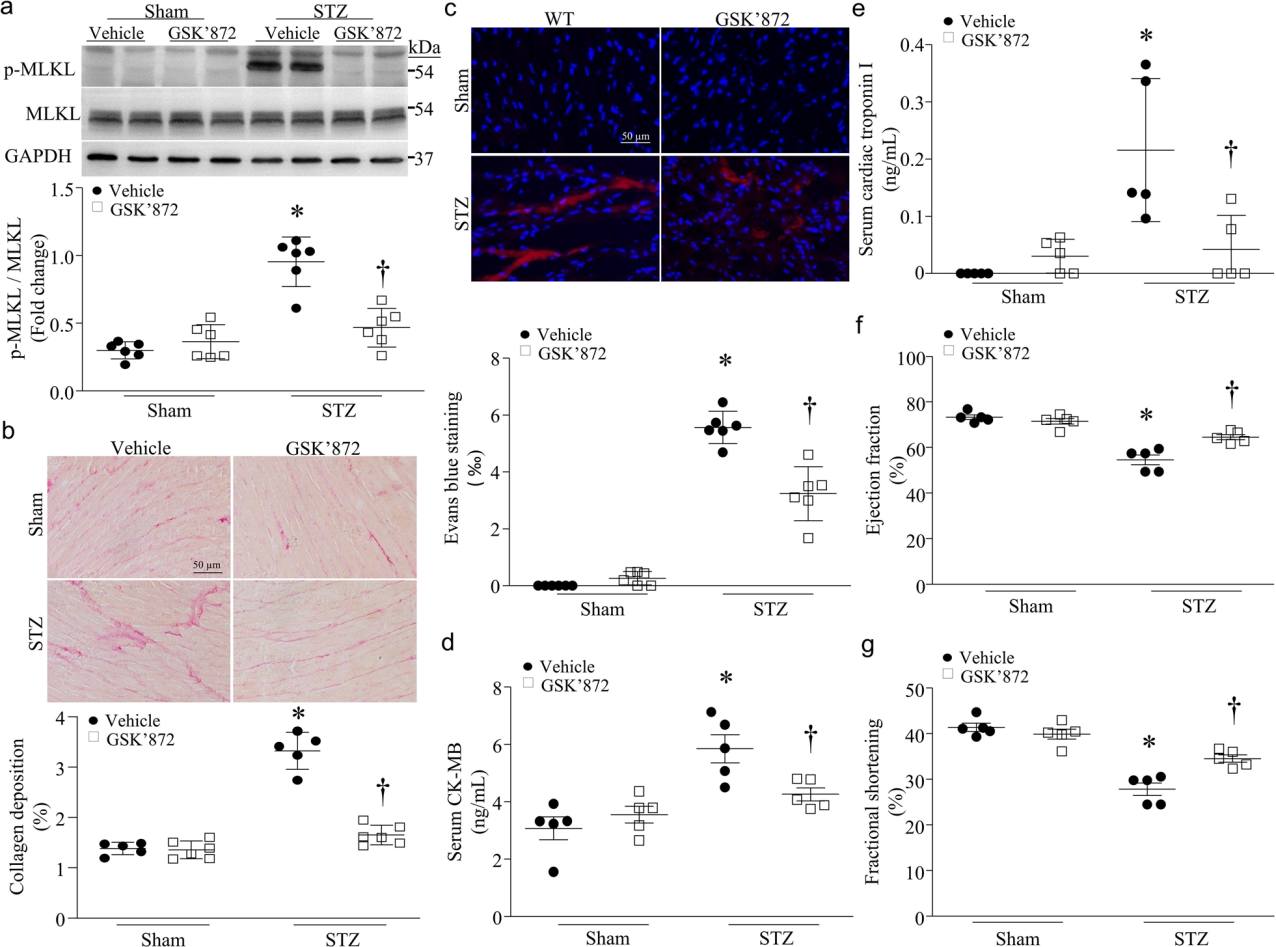
Effects of GSK’872 in streptozocin (STZ)-induced diabetic hearts. Wild-type mice were rendered diabetic by STZ injection and after the induction of diabetes, GSK’872 was administrated for a total of 2 months. **a** The levels of phosphorylated MLKL (p-MLKL) were analyzed by western blot in the heart. Upper panel: a representative western blot from 2 out of 6 different hearts in each group for p-MLKL and total MLKL; Bottom panel: quantification of p-MLKL relative to total MLKL. **b** Myocardial fibrosis was assessed by collagen deposition in the heart. Upper panel: representative micro-photograph for collagen deposition in the heart (red); Bottom panel: quantification of collagen deposition. **c** Necrotic cell death in the heart was determined by Evans blue staining assay. Upper panel: representative micro-photograph for necrotic cells in the heart; Bottom panel: quantification of necrotic cell death. **d** and **e** Biomarkers of myocardial injury: serum cardiac troponin I (**e**) and CK-MB (**d**) and. **f** and **g** Echocardiographic analysis for myocardial function. Data are mean ± SD, n = 5–6. * *P* < 0.05 versus Sham + Vehicle and ^†^*P* < 0.05 versus STZ + Vehicle (ANOVA)

To determine the role of RIPK3 in MLKL phosphorylation and diabetic cardiomyopathy, we induced type-1 diabetes in RIPK3 knockout and wild-type mice by STZ injection. Two months after STZ injection, no death was observed. MLKL phosphorylation was increased in wild-type mouse hearts; however, its level was much lower in RIPK3 knockout mouse hearts (Fig. 4a), indicating that RIPK3 mediates MLKL activation in diabetic hearts. Deletion of RIPK3 prevented cardiomyocyte necroptosis as determined by the Evans blue staining (Fig. 4b), attenuated myocardial dysfunction as evidenced by decreased FS% and EF% (Fig. 4c, d, Additional file 1: Table S1), reduced myocardial collagen deposition (Fig. 4e), and decreased serum biomarkers of myocardial injury (CK-MB and troponin I) in STZ-injected mice (Fig. 4f, g). To examine if targeting RIPK3 is a therapeutic approach to reduce diabetic cardiomyopathy, we gave GSK’872, a pharmacological inhibitor of RIPK3, to STZ-injected mice right after induction of hyperglycemia for a total of 2 months. Similarly, pharmacological inhibition of RIPK3 prevented MLKL phosphorylation (Fig. 5a), attenuated myocardial collagen deposition (Fig. 5b), reduced cardiomyocyte necroptosis (Fig. 5c) and the levels of CK-MB and troponin I in sera (Fig. 5d, e), and improved myocardial function in STZ-injected mice (Fig. 5f, g, Additional file 1: Table S2). Neither RIPK3 deletion nor GSK’872 had any effects on blood glucose and body weight in sham and diabetic mice (Additional file 1: Tables S1 and S2). Under normal conditions, RIKP3 knockout or GSK’872 did not affect myocardial function in mice (Additional file 1: Tables S1 and S2).

### Deletion of MLKL diminishes cardiomyocyte necrosis and reduces cardiomyopathy in STZ-injected mice

**Fig. 6:**
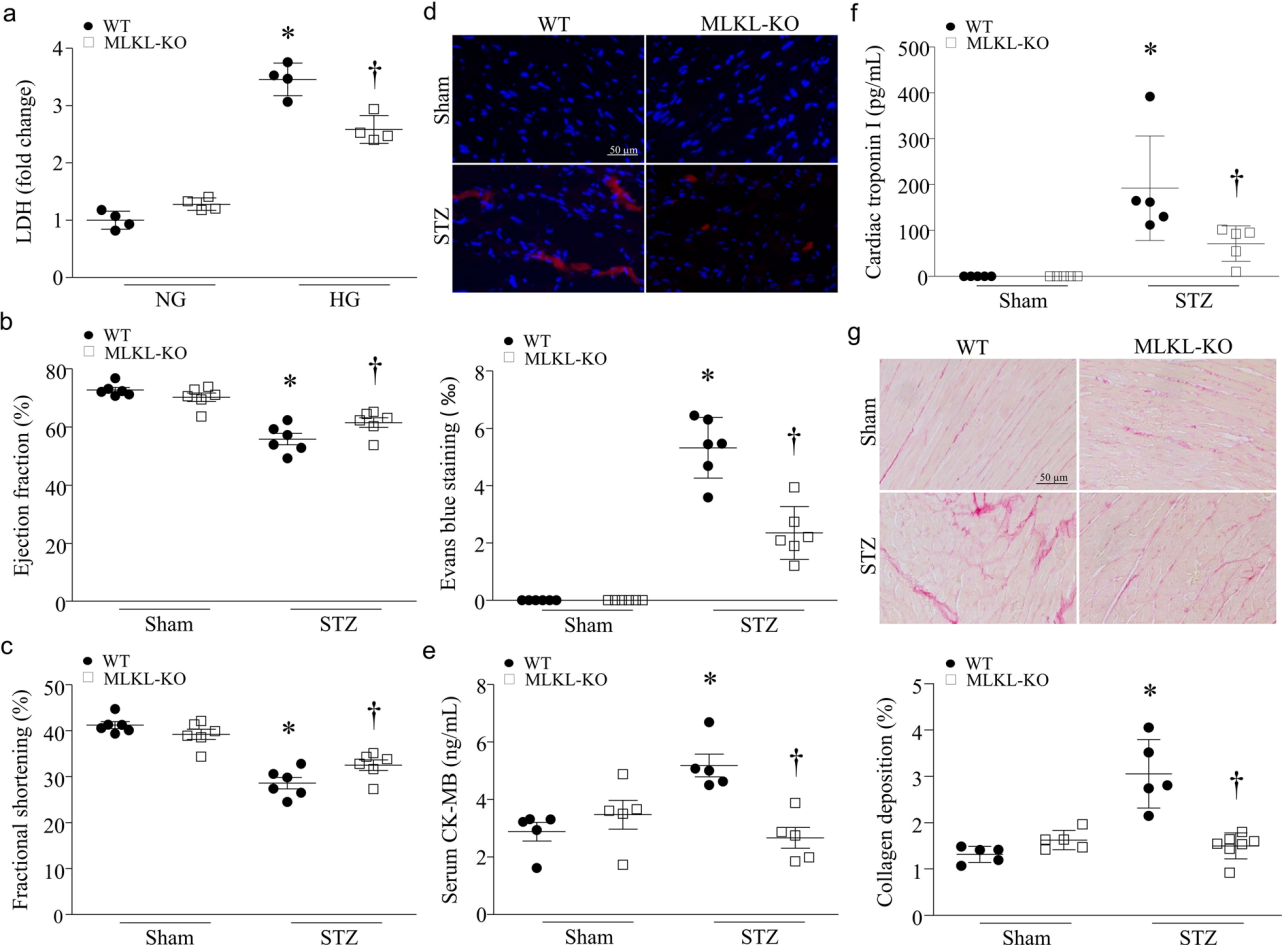
Effects of MLKKL knockout in streptozocin (STZ)-induced diabetic hearts. **a** Adult cardiomyocytes isolated from MLKL knockout (MLKL-KO) and wild-type mice (WT) were incubated with high glucose or Mannitol (25 mmol/L) for 24 h. LDH was measured in culture medium. **b**–**g** MLKL-KO and WT mice were rendered diabetic by STZ injection. **b** and **c** Echocardiographic analysis for myocardial function. **d** Necrotic cell death in the heart was determined by Evans blue staining assay. Upper panel: representative micro-photograph for necrotic cells in the heart; Bottom panel: quantification of necrotic cell death. **e** and **f** Biomarkers of myocardial injury: serum CK-MB (**e**) and cardiac troponin I (**f**). **g** Myocardial fibrosis was assessed by collagen deposition in the heart. Upper panel: representative micro-photograph for collagen deposition in the heart (red); Bottom panel: quantification of collagen deposition. Data are mean ± SD, n = 5–6. *P < 0.05 versus Sham + WT and ^†^*P* < 0.05 versus STZ + WT (ANOVA)

To determine the role of MLKL, we first incubated MLKL-knockout and wild-type mouse cardiomyocytes with high glucose or mannitol. Twenty-four hours later, the LDH release assay revealed that the LDH level in culture medium was much higher in wild-type compared with MLKL-knockout cardiomyocytes after high glucose incubation (Fig. 6a), suggesting a protective effect of MLKL deficiency in high glucose-stimulated cardiomyocytes. We then induced type-1 diabetes in MLKL-knockout and wild-type mice by injection of STZ. Two months after STZ injection, no death was observed and echocardiography revealed that myocardial function was relatively preserved in MLKL knockout mice (Fig. 6b, c, Additional file 1: Table S3). Deletion of MLKL prevented cardiomyocyte necrosis determined by the Evans blue assay (Fig. 6d) and attenuated CK-MB and troponin I levels in sera (Fig. 6e, f), reduced myocardial collagen deposition in STZ-injected mice (Fig. 6g). Blood glucose and body weight were comparable between MLKL knockout and wild-type mice under either normal or diabetic conditions. Deletion of MLKL did not change myocardial function and fibrosis in sham animals (Fig. 6g, Additional file 1: Table S4).

### Knockdown of MLKL attenuates cardiomyocyte necroptosis in akita type-1 diabetic mice

**Fig. 7:**
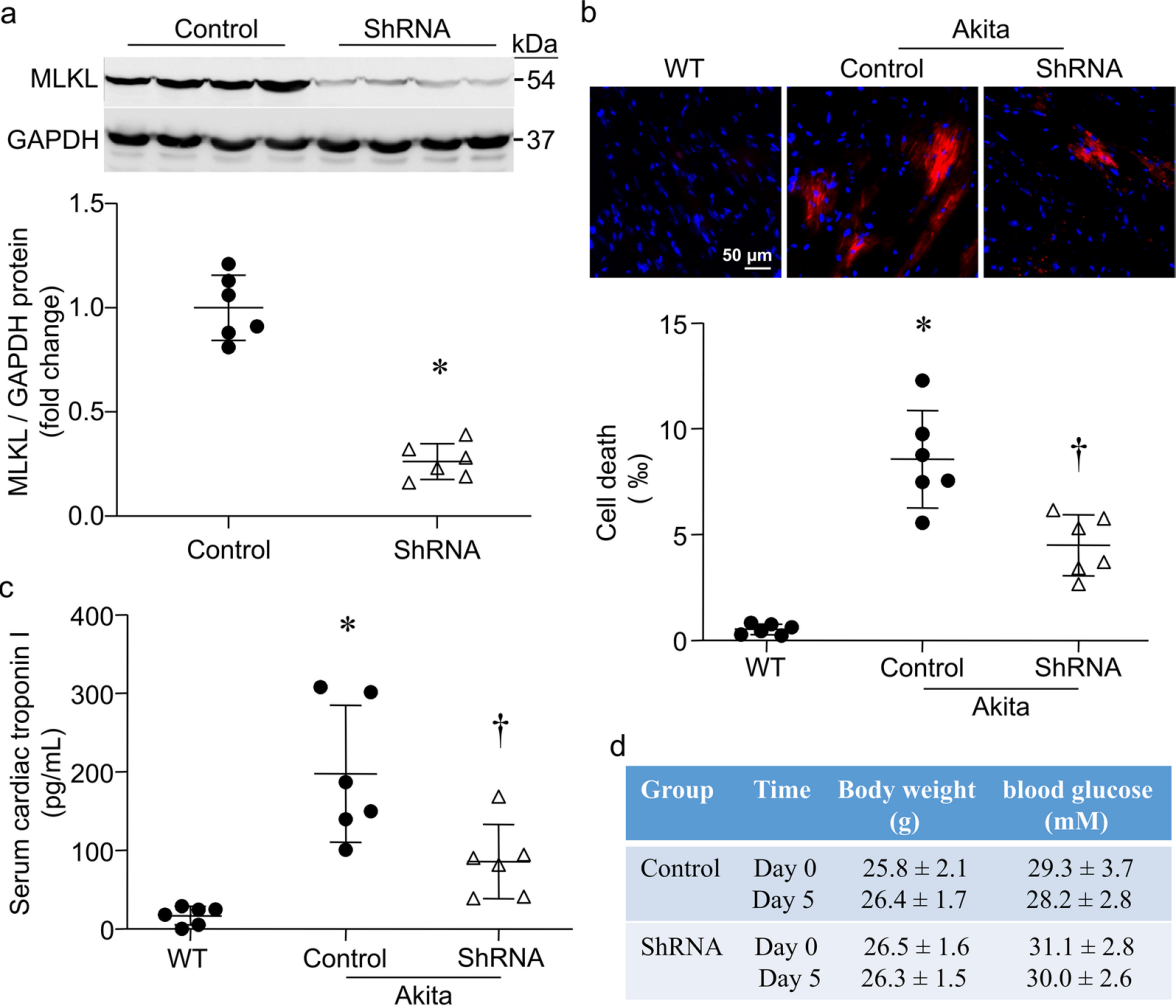
Effects of MLKL knockdown in streptozocin (STZ)-induced diabetic hearts. Akita mice received two separate doses of shRNA for MLKL on day 0 and day 3, respectively. Wild-type mice (WT) served as Sham. Control Akita mice received same amounts of an empty plasmid. On day 5, mice were sacrificed. **a** The levels of MLKL protein were analyzed by western blot in the heart. Upper panel: a representative western blot from 4 out of 6 different hearts in each group for MLKL and GAPDH; Bottom panel: quantification of MLKL relative to GAPDH protein. **b** Necrotic cell death in the heart was determined by Evans blue staining assay. Upper panel: representative micro-photograph for necrotic cells in the heart; Bottom panel: quantification of necrotic cell death. **c** Biomarker of myocardial injury: serum cardiac troponin I. **d** Body weight and blood glucose levels in Akita mice on day 0 and day 5. Data are mean ± SD, n = 6. *P < 0.05 versus Control and ^†^*P* < 0.05 versus Control + Akita (t test and ANOVA)

To provide further evidence to support the role of MLKL in cardiomyocyte necroptosis in diabetes, we knocked down MLKL in akita type-1 diabetic mouse hearts by systemic delivery of shRNA against MLKL (*p*shMLKL). An empty plasmid served as a control. Delivery of shRNA reduced the levels of MLKL protein in akita mouse hearts, indicating successful knockdown of MLKL (Fig. 7a). Akita mice exhibited cardiomyocyte necrosis (Fig. 7b), which was correlated with an increase in serum cardiac troponin I levels (Fig. 7c). Knockdown of MLKL in the heart reduced cardiomyocyte necrosis and attenuated serum cardiac troponin I levels in akita mice (Fig. 7b, c). However, knockdown of MLKL did not change body weight and blood glucose levels in akita mice (Fig. 7d).

### Anti-diabetic drugs inhibit necroptosis in cardiomyocytes under diabetic conditions

**Fig. 8:**
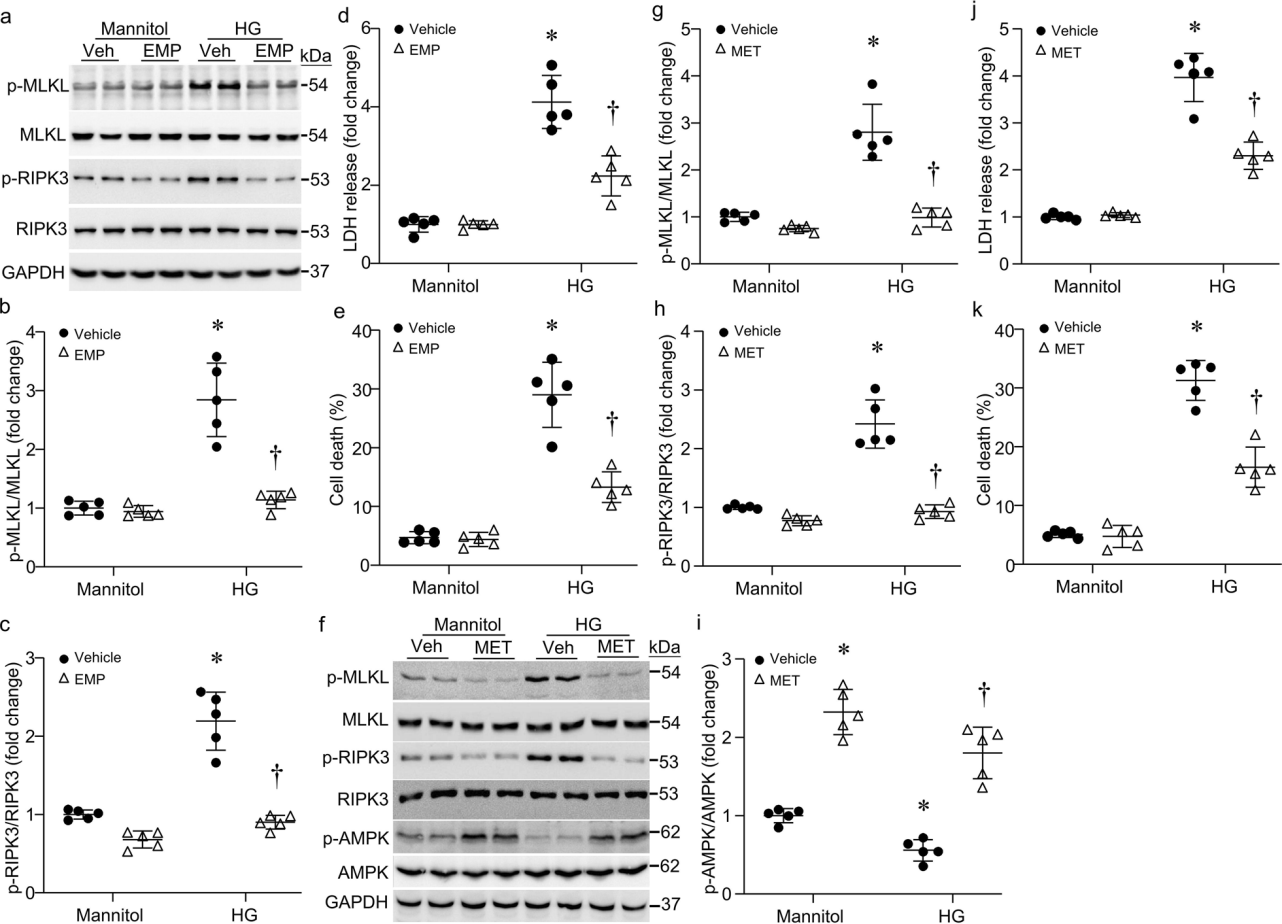
Effects of anti-diabetic drugs on necroptosis in cardiomyocytes. Adult mouse cardiomyocytes were incubated with high glucose (HG) or mannitol (25 mmol/L) in the presence of empagliflozin (EMP, 1 µmol/L), metformin (MET, 500 µmol/L) or Vehicle (Veh) for 24 h. **a** Representative western blots for phosphorylated RIPK3 (p-RIPK3) and MLKL (p-MLKL) and their total proteins as well as GAPDH. **b** and **c**; Quantification of p-RIPK3 and p-MLKL relative to their total proteins, respectively. **d** and **j** LDH was measured in culture medium. (e and k) Cell death was assessed by annexin V staining. **f** Representative western blots for p-RIPK3, p-MLKKL, phosphorylated AMPK (p-AMPK) and their total proteins as well as GAPDH. **g**–**i** Quantification of p-MLKL, p-RIPK3 and p-AMPK relative to their total proteins, respectively. Data are mean ± SD, n = 5 different cultures. *P < 0.05 versus Mannitol + Veh and ^†^*P* < 0.05 versus HG + Veh (ANOVA)

We then examined if anti-diabetic drugs have any effects on cardiomyocyte necroptosis under diabetic conditions. Empagliflozin is an inhibitor of sodium-glucose cotransporter-2 and thus used as an anti-diabetic drug to treat adult patients with type-2 diabetes [38]. It was also reported to help prevent heart failure [39]. Adult cardiomyocytes were incubated with high glucose or mannitol (25 mmol/L) in the presence of empagliflozin (1 µmol/L, Cedarlane, Burlington, Ontario, Canada) [40] or vehicle for 24 h. Co-incubation with empagliflozin significantly reduced high glucose-induced levels of phosphorylated RIPK3 and MLKL in cardiomyocytes (Fig. 8a–c), suggesting its inhibitory effect on necroptosis. This was supported by LDH release and annexin V staining assays. As shown in Fig. 8d, e, high glucose induced LDH release and increased the cell death, both of which were attenuated by empagliflozin. Similarly, incubation with metformin (500 µmol/L, Cedarlane, Burlington, Ontario, Canada), a well-established anti-diabetic drug in clinic, provided a similar inhibitory effect on necroptosis in high glucose-induced cardiomyocytes (Fig. 8f–h, j, k). Consistently, metformin, a well-established activator of AMPK increased the levels of phosphorylated AMPK, indicative of its activation, and prevented high glucose-induced reduction of AMPK in cardiomyocytes (Fig. 8f, i). These results strongly suggest that in addition to their anti-diabetic effects, empagliflozin and metformin have a direct inhibitory effect on cardiomyocyte necroptosis under diabetic conditions.

## Discussion

Cardiomyocyte death is the fundamental mechanism contributing to cardiac complications of diabetes as loss of cardiomyocytes triggers the cascade of adverse myocardial remodeling that leads to heart failure [7]. Two forms of cell death (i.e. apoptosis [8, 9, 11–13] and pyroptosis [10, 14, 15]) have been implicated in cardiac complications of diabetes. Recent studies have suggested that necroptosis may also play a role in cardiac complications of diabetes. In H9c2 cells, high glucose was reported to increase the protein levels of the necroptosis signaling RIPK1/RIPK3/MLKL and inhibition of RIPK1 with necrostatin-1 reduced high glucose-induced cell death [22, 41, 42]. High glucose also induced an increase in the protein levels of RIPK1/RIPK3/MLKL signaling in neonatal rat cardiomyocytes, which were associated with cell death [23, 24, 26]. These in vitro studies have engaged the necroptosis signaling in high glucose-induced cardiomyocyte death. In animal models of diabetes including STZ injection-induced type-1 diabetic mice [27], high fat diet-induced pre-diabetic rats [25], high fat diet plus STZ-induced type-2 diabetic rats [43] and db/db type-2 diabetic mice [27], the protein levels of total and/or phosphorylated RIPK1/RIPK3/MLKL were elevated in the heart, suggesting a potential role of necroptosis in cardiac complications of diabetes. Indeed, this was supported by a most recent study which showed that deletion of RIPK3 alleviated myocardial injury and dysfunction in STZ-injected mice [27]. However, cardiomyocyte necroptosis in diabetic hearts has not been well characterized. As we showed that RIPK3 and MLKL are activated in three different models of type-1 diabetes including STZ-induced mouse and monkey as well as Akita mice, which are associated with an elevation of cardiac troponin I in serum, a well-established biomarker of cardiomyocyte injury, we identified necroptotic cardiomyocytes in the heart of both STZ-induced type-1 diabetic mice and Akita mice by double staining of necrosis (Evans blue staining) and phosphorylated MLKL while cardiomyocytes were visualized by staining cardiac troponin I. Following the induction of necroptosis, MLKL phosphorylation initiates its oligomerization, membrane translocation and membrane disruption [44]. In line with this, we showed MLKL oligomerization in diabetic hearts and our immunofluorescent confocal microscopic analysis demonstrated membrane punctate staining of phosphorylated MLKL in diabetic hearts. Importantly, we provided in vivo evidence that cardiomyocyte necroptosis is present in type-1 diabetic hearts and that inhibition of cardiomyocyte necroptosis by targeting RIPK3 or MLKL attenuates myocardial collagen deposition and heart dysfunction in type-1 diabetic mice. The attenuation of myocardial collagen deposition may result from a reduction of cardiomyocyte necroptosis in diabetic hearts as loss of cardiomyocytes is usually replaced by fibroblasts. In adult mouse cardiomyocytes, using a combination of approaches including deletion of RIPK3, deletion of MLKL and pharmacological inhibitor of RIPK3 we confirmed that diabetic condition (i.e. high glucose) induces necroptosis. Thus, our data demonstrate that in addition to apoptosis and pyroptosis, cardiomyocyte necroptosis also makes important contribution to the pathogenesis of type-1 diabetic cardiomyopathy and represents a target for cardiac protection in diabetes. Although the current study was focused on type-1 diabetes, we speculate that cardiomyocyte necroptosis may also play a role in cardiac pathology of type-2 diabetes as the necroptosis signaling RIPK3 and MLKL were activated in the heart of db/db type-2 diabetic mice [27] and high fat diet plus STZ-induced type-2 diabetic rats [43]. Nevertheless, future studies are warranted to clarify the role of necroptosis and its RIPK3/MLKL signaling in cardiac pathology using type-2 diabetic models.

It is well known that the phosphorylation of RIPK3 by RIPK1 results in MLKL activation during necroptotic cell death in many cell types [44]. In cardiomyocytes, however, recent studies indicated that the signaling pathways downstream of RIPK3 may be also independent of MLKL in mediating necroptosis [17, 28]. An important finding of the current study is that MLKL is required for RIPK3-mediated necroptosis in type-1 diabetic cardiomyopathy. Several lines of evidence support this conclusion. First, RIPK3 and MLKL were activated concomitantly in high glucose-induced cardiomyocytes and diabetic hearts. Second, inhibition of RIPK3 by gene deletion or a pharmacological inhibitor blocked MLKL activation in cardiomyocytes and mouse hearts under diabetic conditions. Third, deletion or knockdown of MLKL attenuated diabetic condition-induced necrosis in cardiomyocytes and mouse hearts. On the other hand, it was reported that necroptosis could be mediated by MLKL but independent of RIPK1/RIPK3 signaling in transhinol A-stimulated lung cancer cells [45]. Additionally, MLKL may be activated through RIPK3-independent pathways to facilitate endosomal protein trafficking and degradation [46], which modulates autophagic flux [47], suggesting a necroptosis-independent function of MLKL. However, the current study suggests that RIPK3 mediates MLKL activation in promoting cardiomyocyte necroptosis under diabetic conditions.

Notably, the current study has several translational implications. First, we provided the first evidence that anti-diabetic drugs, empagliflozin and metformin, prevented high glucose-induced necroptosis in cardiomyocytes, supporting a direct beneficial effect of these drugs on cardiac complications of diabetes in addition to their anti-diabetic effects. The anti-necroptotic effect of metformin might be associated with AMPK activation as it prevented high glucose-induced reduction of AMPK activation in cardiomyocytes while AMPK was reported to inhibit RIPK1-RIPK3 complex formation [48]. The anti-necroptotic effect of empagliflozin is currently unknown in cardiomyocytes under diabetic conditions but merits further investigation. Second, the use of a monkey model of type-1 diabetes provided translational evidence that the necroptosis signaling RIPK3 and MLKL are activated in diabetic hearts and may represent new therapeutic targets for cardiac complications of diabetes. Third, pharmacological inhibition of RIPK3 attenuated cardiomyocyte death and cardiac pathology in a mouse model of type-1 diabetes, indicating the potential of pharmacological inhibition of necroptosis signaling as a useful therapeutic intervention for cardiac complications of diabetes. Lastly, shRNA-mediated knockdown of MLKL provided an in vivo potential gene therapy to reduce diabetic cardiomyopathy by targeting necroptosis signaling. However, there are some limitations. For example, global knockout of RIPK3 and MLKL or systemic delivery of shRNA for MLKL may have confounding effects on other organs. Nevertheless, our in vitro study using cultured adult cardiomyocytes substantiates the role of RIPK3/MLKL in cardiomyocyte necroptosis. It is important to mention that deletion of RIPK3 and MLKL did not affect blood glucose level in normal and diabetic mice.

In summary, we have provided evidence that cardiomyocyte necroptosis plays an important role in mediating cardiac pathology of type-1 diabetes and that MLKL-mediated necroptosis contributes to diabetic cardiomyopathy. Thus, targeting necroptosis may serve as a cardioprotective strategy for cardiac complications of diabetes.

## Supplementary Information

**Additional file 1.** Supplementary Figures and Tables.

## Data Availability

The datasets used and/or analysed during the current study are available from the corresponding author on reasonable request.

## Abbreviations

MLKL: Mixed lineage kinase domain-like pseudokinase 1
RIPK3: Receptor interacting protein kinase 3
STZ: Streptozocin
EBD: Evans blue dye
OCT: Optical cutting temperature
CK-MB: Creatine kinase MB
LV: Left ventricle
FS: Fractional shortening
EF: Ejection fraction
LDH: Lactate dehydrogenase
PI: Propidium iodide
HRP: Horseradish peroxidase
