# Supplementary material for: MLKL-mediated necroptosis is a target for cardiac protection in mouse models of type-1 diabetes
Source: Cardiovasc Diabetol. 2022 Aug 27;21:165. doi: 10.1186/s12933-022-01602-9 (PMC9420252; doi:10.1186/s12933-022-01602-9)
Supplement: Supplementary file 1 — Additional file 1. Supplementary Figures and Tables. [file 12933_2022_1602_MOESM1_ESM.docx]

**Electronic Supplementary Materials (ESM)**


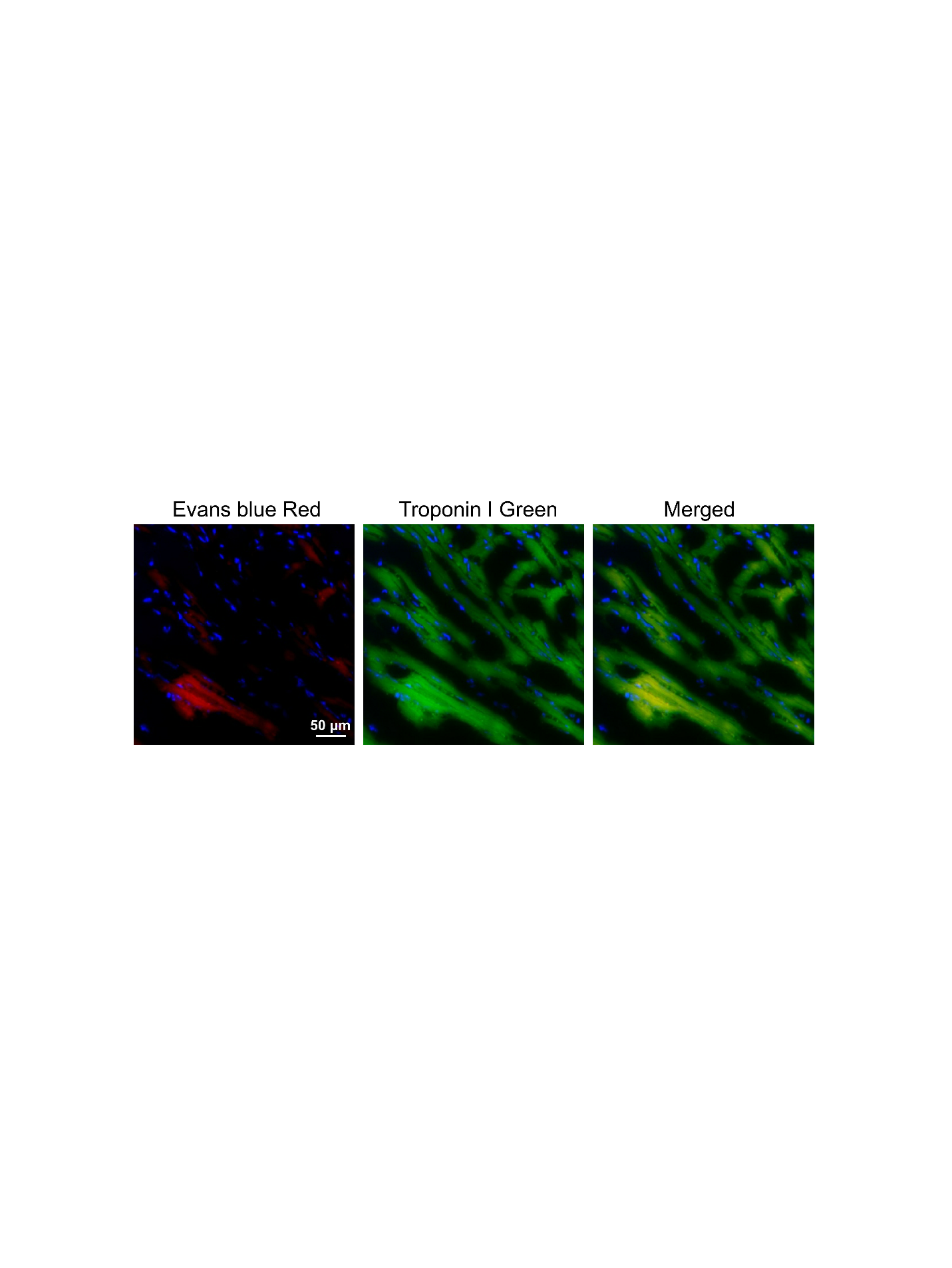
**ESM Fig. 1s. Identification of Evans blue positive cardiomyocytes in STZ-injected mouse hearts.** Frozen sections of STZ-injected mouse hearts were subjected to immuno-fluorescent staining for troponin I (green). Representative micro-photograph shows co-localization of Evans blue positive staining (red) with troponin I staining (green) in STZ-injected mouse hearts. Nuclei were counteracted by Hoechst 33342 (blue).


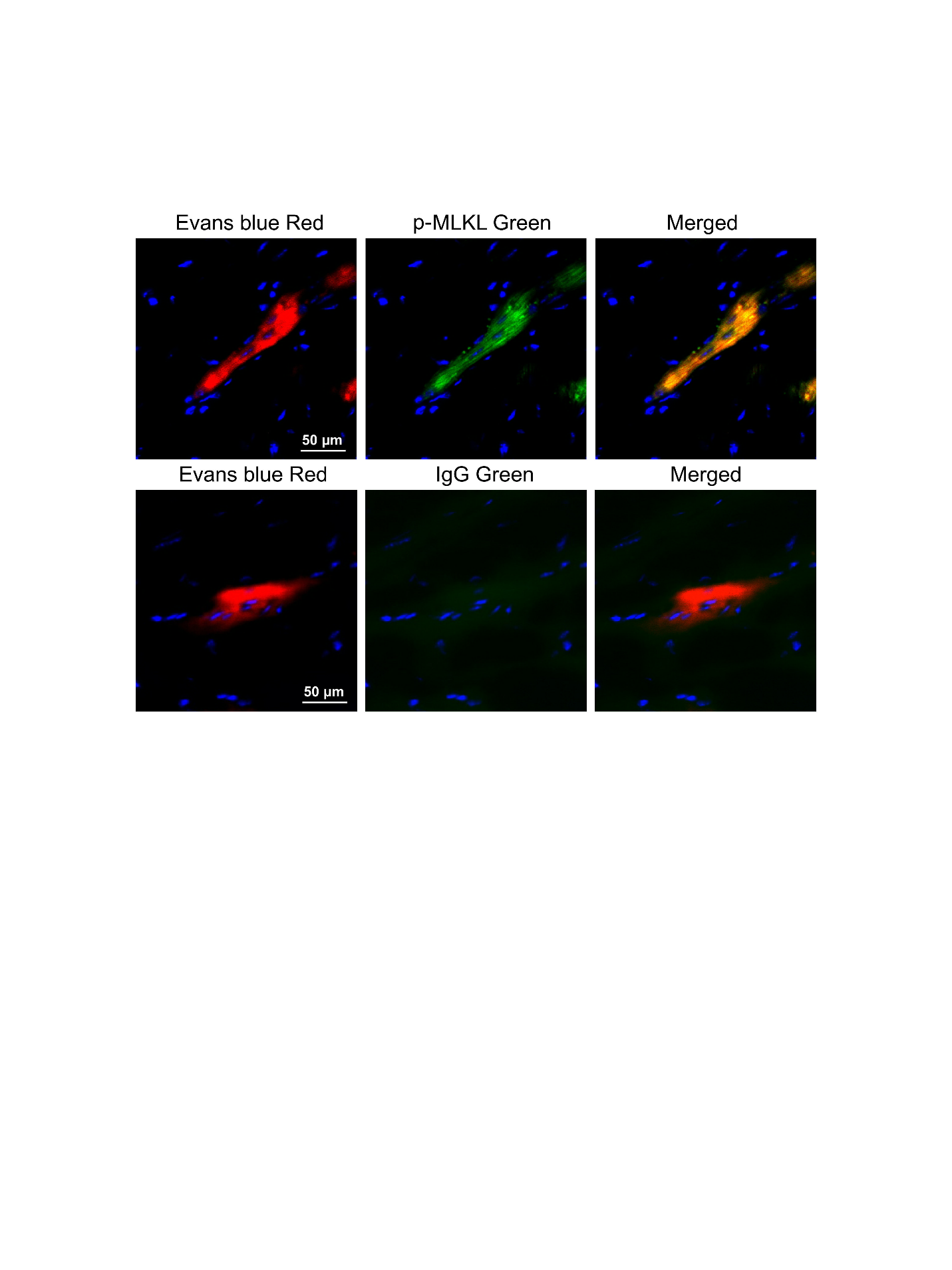


**ESM Fig. 2s. Localization of phosphorylated MLKL positive necrotic cell death in the heart.** Frozen sections of STZ-injected mouse hearts were subjected to immuno-fluorescent staining. Upper panel: representative micro-photograph shows co-localization of Evans blue positive staining (red) with phosphorylated MLKL (green) in STZ-injected mouse hearts. Bottom panel: representative micro-photograph shows that Evans blue positive necrotic cells are not stained when antibodies against phosphorylated MLKL were replaced by a control IgG antibody. Nuclei were counteracted by Hoechst 33342 (blue).


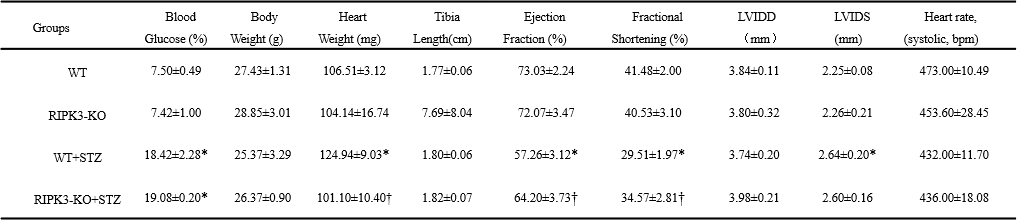


**ESM Table-1: Metabolic parameters and echocardiographic analysis**

(STZ: Streptozocin; bpm: beats per minute. **P* < 0.05 *versus* WT and †*P* < 0.05 *versus* WT+STZ)


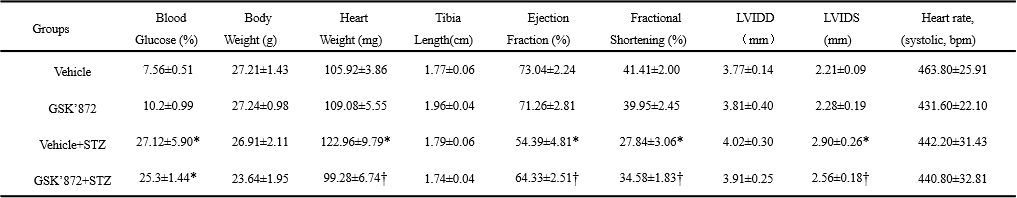


**ESM Table-2: Metabolic parameters and echocardiographic analysis**

(STZ: Streptozocin; bpm: beats per minute. **P* < 0.05 *versus* Vehicle and †*P* < 0.05 *versus* Vehicle+STZ)


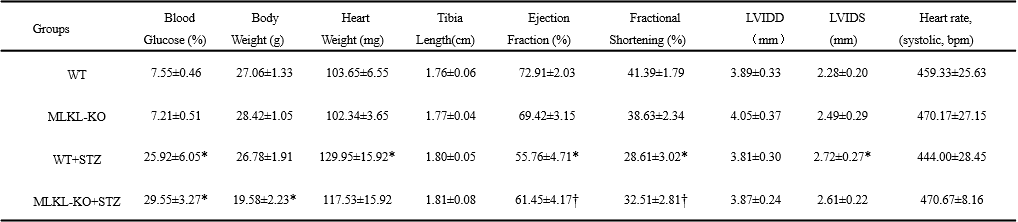


**ESM Table-3: Metabolic parameters and echocardiographic analysis**

(STZ: Streptozocin; bpm: beats per minute. **P* < 0.05 *versus* WT and †*P* < 0.05 *versus* WT+STZ)
